# Genomic Analysis of the Evolution and Global Spread of Hyper-invasive Meningococcal Lineage 5

**DOI:** 10.1016/j.ebiom.2015.01.004

**Published:** 2015-01-13

**Authors:** Odile B. Harrison, James E. Bray, Martin C.J. Maiden, Dominique A. Caugant

**Affiliations:** a Department of Zoology, University of Oxford, Oxford OX1 3PS, UK; b WHO Collaborating Centre for Reference and Research on Meningococci, Norwegian Institute of Public health, P.O. Box 4404 Nydalen, NO-0403 Oslo, Norway; c Department of Community Medicine, Faculty of Medicine, University of Oslo, P.O. Box 1130 Blindern, NO-0318 Oslo, Norway

**Keywords:** *Neisseria meningitidis*, Serogroup B, Genome sequencing, Evolution

## Abstract

**Background:**

The predominant model for bacterial pandemics is the emergence of a virulent variant that diversifies as it spreads in human populations. We investigated a 40-year meningococcal disease pandemic caused by the hyper-invasive ET-5/ST-32 complex.

**Methods:**

A global collection of *Neisseria meningitidis* isolates dating from 1969 to 2008 was whole genome sequenced (WGS) and analysed using a gene-by-gene approach at http://pubmlst.org/neisseria.

**Findings:**

Analysis of WGS data identified a ‘Lineage 5 pan genome’ of 1940 genes, 1752 (92%) of which were present in all isolates (Lineage 5 ‘core genome’). Genetic diversity, which was mostly generated by horizontal gene transfer, was unevenly distributed in the genome; however, genealogical analysis of diverse and conserved core genes, accessory genes, and antigen encoding genes, robustly identified a star phylogeny with a number of sub-lineages. Most European and American isolates belonged to one of two closely related sub-lineages, which had diversified before the identification of the pandemic in the 1970s. A third, genetically more diverse sub-lineage, was associated with Asian isolates. Several isolates had acquired DNA from the related gonococcus.

**Interpretation:**

These data were inconsistent with a single point of origin followed by pandemic spread, rather suggesting that the sub-lineages had diversified and spread by asymptomatic transmission, with multiple distinct strains causing localised hyperendemic outbreaks.

## Highlights

• Genomic analysis of hyper-invasive *N. meningitidis* lineage 5 distinguishing sub-lineages and localised outbreaks.
• Identification of a gonococcal conjugative plasmid consistent with horizontal genetic transfer.
• Discovery of a distinct type 4 secretion system previously unidentified in *Neisseria*.
• Gene-by-gene analysis of conserved and diverse loci providing essential tools in serogroup B vaccine development.

## 1 Introduction

*Neisseria meningitidis*, a Gram negative diplococcal bacterium, is normally a commensal resident of the oropharynx of a high percentage (10–30%) of the human population, very occasionally, causing life-threatening meningitis and septicaemia (Caugant and Maiden, 2009). The only well-established virulence factor of *N. meningitidis* is the polysaccharide capsule, which mediates resistance to complement-mediated lysis and opsonophagocytosis. Based on biochemical composition as well as genetic analysis, 12 serogroups have been described of which 6 (serogroups A, B, C, W, Y and, X) are associated with most disease worldwide (Harrison et al., 2013). Capsule polysaccharide conjugate vaccines have been successfully used to induce protective immunity against *N. meningitidis* serogroups A, C, W and, Y. However, due to similarities between the serogroup B polysaccharide and human glycoprotein structures, no such vaccine targeting this serogroup is available.

The genetic diversity and population structure of the species have been elucidated by the use of two related methods, multilocus enzyme electrophoresis (MLEE) starting in the early 1980s and multilocus sequencing typing (MLST) at the end of the 1990s (Caugant et al., 1986a, Maiden et al., 1998). Both methods assess genetic variation among isolates by indexing their whole genome through a small subset of representative housekeeping genes. With the availability of high-throughput Sanger DNA sequencing, MLEE was replaced by MLST, which presents the additional advantage of being fully portable through an Internet database (www.pubmlst.org/neisseria). Both methods produce equivalent data and the basic features of meningococcal populations first elucidated by MLEE were confirmed by MLST.

Of the thousands of genotypes, distinguished by MLEE and MLST most are rarely, if ever, associated with disease, in contrast to the handful of clonal complexes responsible for epidemics or even pandemics (Yazdankhah et al., 2004). Many serogroup B outbreaks since the 1970s have been caused by *N. meningitidis* isolates belonging to the ST-32 clonal complex, previously designated electrophoretic type (ET)-5 complex. ET-5 was first identified from a case of serogroup B meningococcal disease in Norway in 1969 (Caugant et al., 1987). In the succeeding years, a hyper-endemic wave of serogroup B meningococcal disease started in Norway with an incidence reaching 8.7 per 100,000 population in 1983, subsequently decreasing over the years to less than 1.0 per 100,000 population in 2000. Similar or closely related clones expressing the same or different major antigenic outer membrane proteins were responsible for high incidence of serogroup B disease in several other European countries in the 1980s and 1990s, as well as outbreaks and epidemics in Latin America, including Cuba, Chile, Brazil, and Argentina (Bygraves et al., 1999, Caugant et al., 1986b, Cruz et al., 1990, Sacchi et al., 1992, Wedege et al., 1995). The ST-32/ET-5 complex has also caused a prolonged outbreak in the Pacific-Northwest of United States in 1993 to 2007 (Diermayer et al., 1999). A long-lasting outbreak in Normandy, France, in the past decade has also been caused by the ST-32 complex (Rouaud et al., 2006). While serogroup B disease is rare in Asia and Africa, the few available serogroup B isolates were also linked to the same clonal complex. Thus, the ST-32 complex caused disease globally over a 40-year period (Caugant et al., 1987).

Complete “finished” genomes for two ST-32 complex isolates, MC58 and H44/76, have been published, the former originating from the United Kingdom in the 1980s and the latter from a case in Norway in 1976 (Tettelin et al., 2000, Piet, 2011). Both isolates have been extensively used in serogroup B vaccine research with MC58 pivotal to the design of the 4CMenB vaccine (BexSero®) through a technique known as reverse vaccinology and H44/76 used in the design of several outer membrane vesicle-based vaccines (Serruto et al., 2012, van der Ley and Poolman, 1992). Serogroup B vaccine research has focussed on surface-expressed proteins which, while being immunogenic, might also be strongly under selection pressure. It is, therefore, essential to elucidate how these vaccine antigens might change over time and during worldwide spread of a hyper-invasive clone.

**Fig. 1:**
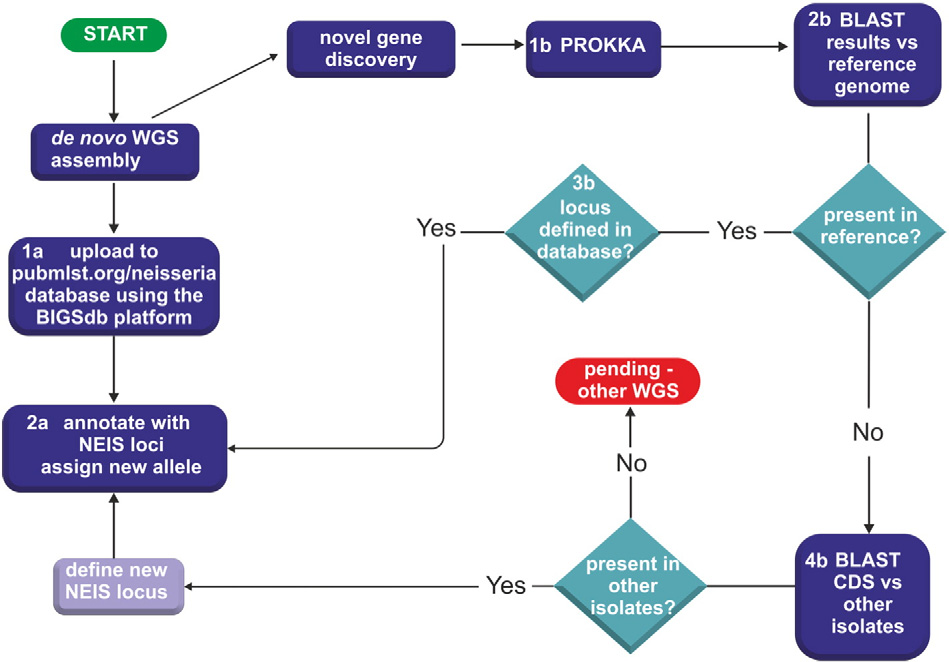
Population annotation pipeline. This pipeline provides a generalizable approach to the curation and annotation of WGS which can be applied to other lineages. It combines the use of: i) the Bacterial Isolate Genome Sequence database (BIGSdb) and, ii) the prokaryotic annotation tool prokka for novel gene discovery. At the time of writing, over 2000 *Neisseria* genes (NEIS loci) had been defined and, once deposited in the database (step 1a), WGS data were automatically annotated with NEIS loci (step 2a). Novel gene discovery used prokka (step 1b) and new genes were screened against reference genomes (2b) as well as NEIS loci defined in the database (3b), thereby eliminating genes which were already being curated. The remaining genes were then checked against WGS data belonging to other isolates enabling the distribution of novel genes to be determined (4b). Before new loci were defined in the database, these were checked in the genome annotation tool, Artemis.

WGS provides a new means to elucidate genomic variation within a clonal complex of *N. meningitidis* and this paper presents a gene-by-gene description of WGS data from a global selection of isolates belonging to the ST-32 clonal complex. A pipeline for the population annotation of WGS has been developed (Fig. 1) combining the use of: i) the Bacterial Isolate Genome Sequence platform (BIGSdb) hosted on the www.pubmlst.org/neisseria database which currently enables the curation of over 2000 *Neisseria* genes and, ii) the prokaryotic annotation tool, prokka for novel gene discovery (Seemann, 2014). Through comparison with reference genomes, the Lineage 5 core genome (Lineage 5 cgMLST) was defined and compared between isolates revealing three distinct clusters of isolates grouping by PorA type within which small localised clusters were also visible. Novel gene discovery identified the Lineage 5 pan genome (Lineage 5 pgMLST) and included type IV secretion systems (T4SS), haptoglobin–haemoglobin receptors associated with iron acquisition, as well as a gonococcal conjugative plasmid.

## 2 Methods

### 2.1 Isolate Collection, WGS and Assembly

Forty-three *N. meningitidis* isolates belonging to clonal complex ST-32, were selected from the strain collection at the WHO Collaborating Centre for Reference and Research on Meningococci in Oslo, Norway. This collection of isolates was chosen to be representative of the 40 year global pandemic forming a baseline dataset for the analysis of this lineage and others. Isolates were retrieved from storage, inoculated onto Columbia horse blood agar and incubated for 24 h at 37 °C in a 5% CO_2_ atmosphere. Prior to DNA extraction, colonies were visually checked for purity. A number of quality controls are carried out following Illumina sequencing to confirm the purity of the samples and to check for contamination. Firstly, assembled genome sizes are verified to ensure they are within the expected *Neisseria* genome range (Table 1) as contaminated samples will contain a distinctly larger genome. Secondly, all samples are scanned for the 53 ribosomal gene proteins which are genus, species as well as clonal complex specific (Jolley et al., 2012a, Jolley and Maiden, 2013). Should a sample be contaminated, conflicting alleles for the ribosomal genes will be apparent. *Neisseria* species can be further identified using the 50 S ribosomal protein L6 (*rplF*) gene (Bennett et al., 2014) and once again, contaminated DNA samples will contain conflicting *rplF* sequence data. The genomes of MC58 (accession numbers in brackets AE002098) and H44/76 (AEQZ00000000), from the UK and Norway respectively, were included as well as isolate, CU385 (AEQJ01000000) (Tettelin et al., 2000, Piet, 2011, Budroni et al., 2011). This gave a final total of 46 isolates analysed in this study.

**Table 1:** List of isolates used.

Isolate (aliases)	Country of Origin	Year of isolation	Strain designation	Sub-lineage	Total assembled bases^a^	Number of contiguous sequences	N50 (bp)^e^
NO-69A (P15)	Norway	1969	B: P1.7,16: F3-3: ST-32	5.2	2,180,518	266	39,354
NO-69B (P28)	Norway	1969	B: P1.7,16: F3-3: ST-32	5.2	2,177,816	259	41,208
US-77 (H172)	USA	1970s	B: P1.7,16: F3-3: ST-32	5.2	2,176,083	268	35,541
CN-74 (2645)	China	1974	B: P1.7,16: F1-7: ST-32	5.1	2,179,027	320	32,925
NO-75 (H355)	Norway	1975	B: P1.19,15: F3-3: ST-32	5.2	2,168,044	256	44,590
NO-76 (H44/76)	Norway	1976	B: P1.7,16: F3-3: ST-32	5.2	2,240,883	1	n/a
DK-76 (25/76)	Denmark	1976	B: P1.19,15: F5-1: ST-2400	5.3	2,171,978	327	30,130
SH-78 (277)^b^	Shanghai	1978	B: P1.7,16: F5-5: ST-1784	5.1	2,240,463	337	37,026
CA-78 (82238)	Canada	1978	B: P1.7,16-2: F1-5: ST-32		2,179,256	266	32,230
JP-79 (58)^c^	Japan	1979	B: P1.7,16: F3-3: ST-32	5.2	2,208,452	343	37,086
CA-79 (79694)	Canada	1979	B: P1.7,16: F1-7: ST-32	5.1	2,173,910	298	30,858
TH-80 (Nimitpol)^d^	Thailand	1980	B: P1.7,16: F3-3: ST-10247	5.1	2,212,441	319	37,283
CL-80 (8733)	Chile	1980	B: P1.7-2,3: F3-1: ST-32		2,150,718	323	29,388
NO-81 (NG080)	Norway	1981	B: P1.7,16: F3-3: ST-32	5.2	2,094,073	294	15,830
NO-82 (NG144/82)	Norway	1982	B: P1.7,16: F3-3: ST-32	5.2	2,088,622	353	10,926
UK-83 (MC58)	UK	1983	B: P1.7,16-2: F1-5: ST-74		2,272,360	1	n/a
CU-83 (CU385)	Cuba	1983	B: P1.19,15: F5-1: ST-33	5.3	2,253,883	47	n/a
NL-84 (BZ83)	Netherlands	1984	B: P1.5-2,10: F5-1: ST-34	5.3	2,078,536	278	14,417
NL-85 (BZ169)^c^	Netherlands	1985	B: P1.5-2,16: F3-3: ST-32		2,176,873	299	27,882
DE-85 (EG329)^c^	East Germany	1985	B: P1.7-1,16: F1-2: ST-32	5.2	2,120,308	336	13,271
NO-85 (NGPB24)	Norway	1985	B: P1.7-2,16-7: F3-3: ST-32	5.2	2,114,016	321	25,396
SP-85A (MA5587)	Spain	1985	B: P1.7,16: F3-3: ST-32		2,180,168	277	30,611
SP-85B (MA5873)	Spain	1985	B: P1.19,15: F1-64: ST-33	5.3	2,164,863	246	34,687
ZA-85 (350)	South Africa	1985	B: P1.19,15: F5-1: ST-10245	5.3	2,175,017	308	36,787
JP-86 (86)	Japan	1986	B: P1.7,16: F1-32: ST-2338	5.1	2,171,256	301	31,703
BR-86 (27/86)	Brazil	1986	B: P1.19,15: F5-1: ST-33	5.3	2,176,357	266	29,904
BR-87 (71/87)	Brazil	1987	B: P1.19,15: F5-1: ST-33	5.3	2,172,645	332	34,952
NO-87 (196/87)^c^	Norway	1987	C: P1.7-2,16-12: F3-3:ST-32	5.2	2,129,967	248	20,187
CL-87 (8680)	Chile	1987	B: P1.7-2,3: F3-1: ST-32		2,088,107	375	16,653
UK-87 (H1100/87)	UK	1987	B: P1.5,2: F5-1: ST-33	5.3	2,175,222	260	38,263
ZA-88 (AO5)	South Africa	1988	B: P1.19,15: F5-1: ST-10246	5.3	2,177,180	269	31,485
BR-89 (84/89)	Brazil	1989	B: P1.7,16: F3-3: ST-32		2,175,899	261	33,291
CU-92A (204/92)^c^	Cuba	1992	B: P1.19,15: F5-1: ST-33	5.3	2,128,705	418	16,437
CU-92B (70/92)^c^	Cuba	1992	B: P1.19,15: F5-1: ST-33	5.3	2,195,929	261	38,836
US-93 (M1037)	USA	1993	B: P1.7,16-33: F3-3: ST-32	5.2	2,169,887	266	38,136
AU-93 (93-N213)^c^	Australia	1993	B: P1.7-2,ND: F1-95: ST-1784	5.1	2,194,952	286	35,794
MA-94A (M50)	Morocco	1994	B: P1.19,15: F5-1: ST-5955	5.3	2,119,902	238	33,079
MA-94B (M64)	Morocco	1994	B: P1.7,16: F3-3: ST-802	5.2	2,135,547	235	47,636
AR-94 (270/94)	Argentina	1994	B: P1.7-2,16: F3-3: ST-1880		2,171,575	330	32,882
NZ-95 (92/30)	New Zealand	1995	B: P1.7,16: F3-3: ST-32	5.2	2,120,815	301	29,473
US-96 (M2528)	USA	1996	B: P1.7,16-20: F3-3: ST-32	5.2	2,167,642	313	36,312
CA-96 (96038)	Canada	1996	B: P1.19,15: F5-1: ST-7783	5.3	2,153 669	325	29,569
CI-98 (MK521/99)	Ivory Coast	1998	B: P1.19,15: F5-1: ST-33	5.3	2,160,095	298	28,426
NO-99 (N24/99)	Norway	1999	C: P1.7,16-51: F3-3: ST-32	5.2	2,123,158	225	49,270
NO-00 (N71/00)	Norway	2000	B: P1.7-2,13-1: F3-3: ST-32	5.2	2,168,635	298	37,113
NO-08 (N20/08)	Norway	2008	B: P1.7,16: F3-3: ST-32	5.2	2,171,498	242	41,776

a Genome size.

b Isolate contains GGI.

c Isolates contain novel virB-like T4SS.

d Isolate contains a gonococcal conjugative plasmid.

e Weighted median statistic indicating that 50% of the entire assembly is contained in contigs equal to or larger than this value.

Genomic DNA was prepared, sequenced and assembled as described previously (Jolley et al., 2012b). The resultant assemblies were uploaded to a database running the BIGSdb platform (www.pubmlst.org/neisseria) and are publicly available (Jolley and Maiden, 2010).

### 2.2 Data Analysis

The BIGSdb software includes ‘autotagger’ and “autodefiner” tools which automatically scan deposited WGS against defined loci identifying alleles greater than or equal to 98% sequence identity. This process runs in the background and automatically updates isolate records with specific allele numbers, marking regions on assembled contiguous sequences (contigs) for any of the defined loci. In addition, PATRIC (Pathosystems Resource Integration Centre http://patricbrc.vbi.vt.edu/portal/portal/patric) and NeMeSys (Rusniok et al., 2009) (http://www.genoscope.cns.fr/agc/microscope/expdata/nemesys) were used to confirm the function and location of loci (Wattam et al., 2014). A pipeline was devised as a guide to the curation and annotation of a population of *N. meningitidis* isolates (Fig. 1).

### 2.3 Identification of the Core and Accessory Genomes

The BIGSdb Genome Comparator tool, implemented within the website was employed to compare isolate WGS data. This tool uses either loci defined within the database or an annotated reference genome as the comparator for analysis. When a reference genome is employed, the coding sequences within the annotation are extracted and compared against the assembled contigs for the isolate genomes under comparison. Unique allele sequences at each locus are designated with an integer starting at 1 (representing identity to the reference sequence). A distance matrix is generated based on the number of variable alleles resolving all of the isolates into networks using the NEIGHBORNET algorithm (Bryant and Moulton, 2004) and a stand-alone instance of SplitsTree4 (Huson and Bryant, 2006).

Isolates were compared using this tool against the reference genomes from MC58 and H44/76 following which 1,752 loci core were found. These were further compared between isolates enabling the identification of: i) identical loci, ii) diverse loci and, iii) conserved loci with the additional option of creating alignments of all loci allowing *p*-distance values to be calculated and underlying sequence differences to be assessed using MEGA v5 (Tamura et al., 2011).

The absence of loci in some genomes was validated by firstly examining the contig on which loci should be located using Artemis and inspecting this region ensuring it had not been replaced by another gene or that this gene was considerably more divergent than the one defined in the database. *Neisseria* genomes contain numerous complex repeat regions which occasionally results in truncated loci located at the end of a contig. This was also checked, confirming whether a locus was in fact present but truncated. Finally, reference sequences containing loci to be examined were created against which short reads were mapped using the Burrows–Wheeler Alignment (bwa) software package and subsequently viewed using Tablet (Li and Durbin, 2009, Milne et al., 2013).

### 2.4 Identification and Annotation of Loci Novel to the ST-32 Clonal Complex

WGS data was analysed using Prokka (Prokaryotic genome annotation software (v1.5.2), www.vicbioinformatics.com) (Seemann, 2014) which uses Prodigal (Hyatt et al., 2010) for automated gene discovery and annotates predicted coding regions using annotations from Pfam (Punta et al., 2012), UniProtKB and user-defined annotated reference genomes.

The translated coding regions of the MC58 genome were added to each Prokka scan in order to differentiate between coding regions previously annotated in MC58 and regions not present in this reference genome. Prokka predicted and annotated a total of 91,968 genes within the 44 isolates, with 7,948 regions identified as absent from the reference genomes. These regions were translated and clustered using blastclus (NCBI BLAST v2.2.10 distribution) using 70% sequence identity and 70% alignment overlap thresholds. The clustering results were manually validated to ensure that all members within each cluster had consistent Prokka-derived annotations. One protein representative was selected from each of the resulting 514 protein clusters. One further sequence search step was necessary to remove 66 coding sequences found to significantly match genes in the second reference genome, H44/76.

The nucleotide sequences of each of the 448 representative genes were mapped onto the genome sequences of each isolate using BLAST search tool within BIGSdb. The genomic context of these novel predicted genes was subsequently visualised using Artemis (Rutherford et al., 2000) and the Prokka-derived annotations were used as a starting point for more detailed manual investigations into the gene functions.

## 3 Results

### 3.1 Genome Assembly and Isolate Characterisation

Isolate designations were used which reflected geographic and temporal origin: the first two letters indicating the country of origin and, the numbers, the last two digits of the year of isolation (Table 1). Genome sequences were obtained using Illumina Sequencing Technology with 54 bp paired-end reads obtained for isolates DK-76, NO-81, NO-82, NL-84, NL-85, DE-85, NO-85, ZA-85, CL-87 and, CU-92A and 100 bp paired-end reads obtained for the remaining isolates (excluding MC58, H44/76 and CU385). The average genome assembly contained 275 contigs with 50% or more of the genome present on a contig ≥31,252 bp in size (Table 1).

MLST curation revealed that the isolates belonged to 14 different sequence types (ST), all part of the central ST-32 clonal complex. Annotation of the PorA (VR1 and VR2) and FetA variable regions showed that 27 isolates contained the PorA VR1 P1.7 and VR2 P1.16 family, along with the FetA VR types F3-3, F1-5, F1-2, F3-1, F5-5, F1-7 and F1-32, the finished genomes from MC58 and H44/76 both belonging to this first set of isolates. A further 11 isolates contained the PorA type P1.19, 15 in combination with the FetA F5-1. Additional FetA types associated with PorA P1.19, 15 were F3-3 or F1-64.

### 3.2 The Lineage 5 Core Genome (cgMLST)

A total of 36 and 24 paralogous loci were identified in the reference genomes, MC58 and H44/76 respectively, including copies of a family of adhesins found to bind to glycolipids on host cells (*mafA*/*mafB* operon) and which include a number of additional hypothetical proteins in each operon, putative large exoproteins involved in haem-utilisation or adhesion and belonging to the ShlA/HecA/FhaA family as well as the iron-repressible repeat-in-toxin (RTX) exoproteins FrpC/FrpA There were, in addition, 25 and 39 transposases listed in MC58 and H44/76. Using current sequencing technologies, paralogous loci do not assemble well resulting in truncated contigs and partial genes. As a result, all of these loci were excluded from further analyses.

**Fig. 2:**
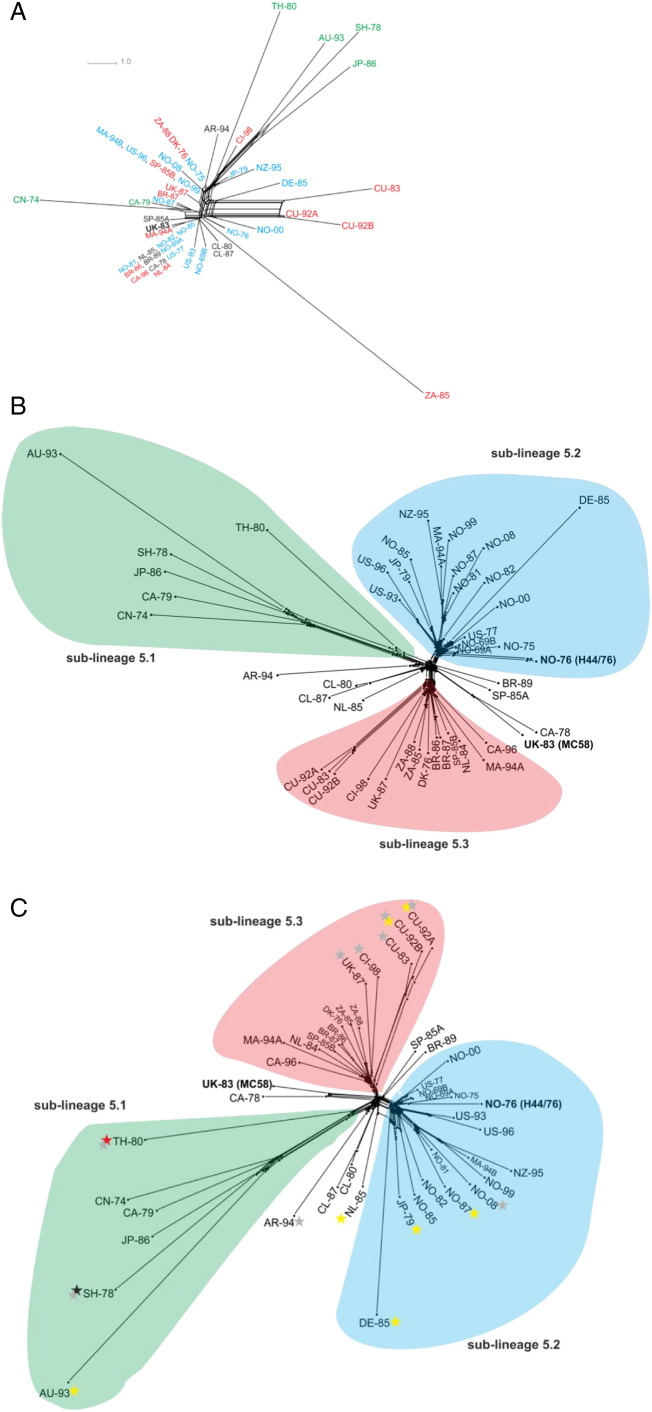
Lineage 5 genealogy. Panel A — genealogical analysis of the 53 ribosomal gene proteins (rMLST). Panel B — genealogical analysis of the 1752 core loci (Lineage 5 cgMLST). Panel C — genealogical analysis of the 1940 loci comprising the Lineage 5 pan-genome (Lineage 5 pgMLST). Sub-lineages are identified by colour with sub-lineage 5.1 isolates in green; sub-lineage 5.2 in blue; sub-lineage 5.3 in red. Black stars denote isolate containing the GGI T4SS; yellow stars represent the *virB*-like T4SS; red star depicts the gonococcal conjugative plasmid; grey stars indicate isolates containing the haptoglobin–haemoglobin HpuAB receptor.

Comparison with the MC58 and H44/76 genomes identified 1,752 loci (95%) core to the Lineage 5 genome. Among these, 214 (12%) loci were invariant and included genes associated with amino acid biosynthesis, energy and DNA metabolism, as well as protein synthesis and fate. There were in addition 110 identical hypothetical proteins (Supplementary Table 1). Genealogical analysis of the 53 ribosomal gene proteins identified 16 identical loci with the remaining loci found to be highly conserved (mean *p*-distance = 0.001) (Supplementary Table 1 and Fig. 2A).

A Lineage 5 core genome scheme was created containing all core 1,752 loci (Lineage 5 cgMLST). Genealogical analysis divided isolates into distinct groups termed sub-lineages, the nomenclature of which was devised to follow on from MLST/MLEE designations (ST-32/ET-5) (Fig. 2B). Sub-lineages could be loosely classified into the “Asian group” (sub-lineage 5.1), the “North European–Norwegian group” which contained isolates with the PorA type P1.7, 16 (sub-lineage 5.2), a “Latin American group” with PorA type P1.19, 15 (sub-lineage 5.3) and, several other isolates which did not fall into either of these groups.

MC58 did not belong to either sub-lineage and clustered separately with an isolate from Canada dating from 1978. Both of these isolates possessed the same PorA and FetA designation (B:P1.7, 16-2: F1-5) although MC58 was ST-74 and CA-78 was ST-32. Isolates from Cuba (CU-83, CU-92 and CU-92B), Chile (CL-80 and CL-87) as well as 2 isolates from Brazil (BR-86 and BR-87) formed distinct clusters within their respective sub-lineages indicative of small localised epidemic clones in circulation in these countries. All of the isolates from Norway, including H44/76, and the United States of America were found in one sub-lineage only (5.2). Isolates from Canada, the Netherlands and the UK were scattered throughout the tree consistent with multiple sub-lineage 5 variants circulating in these countries.

### 3.3 Diversity in the Lineage 5 Core Genome

**Fig. 3:**
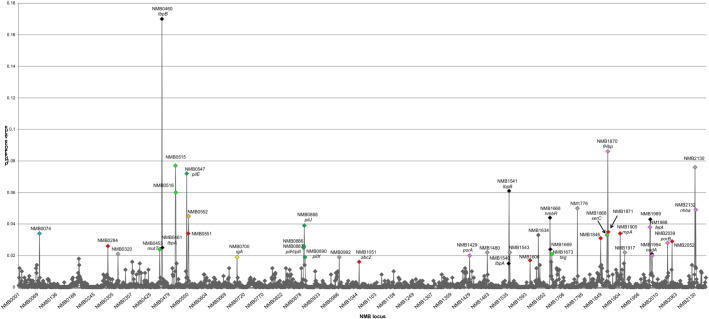
*p*-distance values among all 1752 core loci and between all isolates. Scatter plot with MC58 NMB loci on the X axis and *p*-distance values on the Y axis. Blue diamonds: capsule locus; red: loci associated with metabolism or house-keeping functions; light grey: hypothetical proteins; light green: DNA replication, recombination and repair; black: iron acquisition; yellow: immune evasion; dark green: pilin biogenesis; light orange: denitrification; dark grey: hypothetical proteins.

A total of 41 loci had *p*-distance values between 0.015 and 0.170, equivalent to or higher than those observed in the vaccine antigens PorA, FetA, fHbp, NadA and Nhba (Supplementary Table 2 and Fig. 3).

The highest *p*-distance values were attributed to the surface exposed lipoproteins, *tbpB* (*p*-distance = 0.170) and *lbpB* (*p*-distance = 0.061) which are associated with iron acquisition and have been shown to display extensive variation (Rokbi et al., 1997, Pettersson et al., 1999) with many of the other receptors implicated in iron acquisition also displaying considerable diversity. In addition, 9 isolates (20%), including CU-83, CU-92A, CU-92B, SH-78, AR-94, UK-87, CI-98, NO-08 and TH-80, contained the haptoglobin–haemoglobin receptors, *hpuAB*, which had not been identified in MC58 or H44/76 (Fig. 2C).

### 3.4 The Lineage 5 Pan Genome (pgMLST)

A total of 47 loci (3%) found in MC58 or H44/76 had a variable distribution among the other isolates (Supplementary Table 3). A Lineage 5 pan-genome scheme (Lineage 5 pgMLST) was created containing the 1,752 core loci, the 47 accessory loci identified from MC58 and H44/76 and an additional 141 loci, absent in MC58 and H44/76, but found following Prokka analysis.

These included 38 loci homologous to a 24.5 megadalton (MDa) conjugative plasmid found in *Neisseria gonorrhoeae* and located on a separate 35 kb contig consistent with horizontal genetic transfer between gonococci and meningococci (Pachulec and van der Does, 2010). Although such plasmids have been well-defined in *N. gonorrhoeae*, these have not been identified in meningococci until now. The plasmid was identified in isolate TH-80 and was organised in modules for replication (Rep), conjugative DNA-transfer (Tra), mating-pair-formation (Trb), stable plasmid inheritance and control (Ctl) however, it lacked the *tetM* module conferring tetracycline resistance (Pachulec and van der Does, 2010). Instead, five of the 16 genes described between the Trb and Tra regions in the *N. gonorrhoeae* conjugative plasmid pEP5289 were found: *yegA*, *zeta 2*, *resA* and *vapD* along with 2 hypothetical genes encoding proteins not yet identified in any protein database including PFAM or sharing identified domains.

**Fig. 4:**
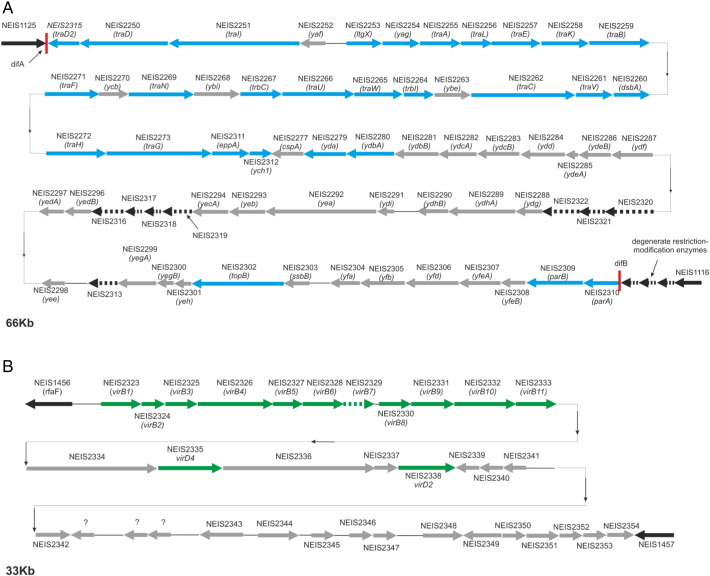
T4SS identified in this study. Two T4SS systems were identified among isolates in this study with the majority of isolates containing the *virB*-like T4SS. Panel A depicts the gonococcal genetic island identified in isolate SH-78. It was 66 kb long and was similar to the meningococcal GGI Mc type 4 identified in *N. meningitidis* alpha 275, a ST-22 serogroup W isolate (Woodhams et al., 2012). It was located between loci NEIS1116 and NEIS1125 which encode uracil-DNA glycosylase and a putative periplasmic protein respectively. Blue arrows depict loci with an essential role in T4SS function while those in grey have putatively unclear roles in T4SS. Panel B depicts the *virB*-like T4SS operon located between NEIS1456 and NEIS1457. It was 33 kb long and was found in isolates DE-85, AU-93, NO-87, JP-79, CU-92A and CU-92B and NL-85. Green arrows represent all of the genes forming part of the *virB* system while those in grey represent additional loci more commonly found in other T4SS. These also included a number of additional loci with unknown functions.

A further 72 loci found in isolate SH-78 formed part of a type IV (T4SS) gonococcal genetic island (GGI) (Fig. 4A). This locus was 66 kb long and was located between genes NEIS1116 (NMB1222) encoding uracil-DNA glycosylase and NEIS1125 (NMB1231) encoding a putative periplasmic protein with three restriction endonucleases located adjacent to NEIS1116. The GGI was not identical to those described in gonococci, but was more homologous to one described in *N. meningitidis* alpha 275 identified as Mc GGI type 4 (Woodhams et al., 2012). An additional T4SS, as yet unidentified in meningococci, was found in seven isolates (Table 1). This contained 29 loci and included all of the 11 *virB* genes associated with the T4SS *virB* operon more commonly found in *Agrobacterium tumefaciens* with the addition of several other open reading frames found in gonococcal GGI, consistent with this being another T4SS. In six of the meningococci here, the operon was located between NEIS1456 (NMB1528) encoding the heptosyltransferase II, *rfaF* and NEIS1457 (NMB1529) encoding a methylated-DNA–protein-cysteine methyltransferase (Fig. 4B). In the seventh isolate, the operon was located before the hypothetical protein, NEIS1669 (NMB0479). Genealogical analysis of the Lineage 5 pan-genome identified a NeighborNet tree comparable to that obtained when examining the 1,752 core loci (Fig. 2B) with the formation of the three sub-lineages 5.1, 5.2 and 5.3 (Fig. 2C).

## 4 Discussion

Persistence of hyper-invasive strain types containing PorA:FetA:clonal complex associations have been described with ST-32 isolates predominantly associated with the antigenic profiles P1.7,16:F3-3 and P1.19,15:F5-1 as found here (Watkins and Maiden, 2012). Data presented here further reveals that these antigenic profiles were congruent with discrete genomic differences leading to the formation of sub-lineages which remained constant throughout all genealogical analyses (Fig. 2B and C). The Lineage 5 core genome (cgMLST) was found to contain 1,752 loci, comprising genes associated with core metabolic functions, nutrient assimilation as well as DNA metabolism, all of which are essential for the successful proliferation of meningococci in the human host. Key virulence determinants such as the capsule locus and genes implicated in iron acquisition were also part of the core genome. Indeed, none of the genes identified in the accessory genome encoded proteins implicated in known key cellular functions (Supplementary Table 3).

Frequent recombination events between isolates from different clonal complexes have been described in core loci including 459 core genes identified by Joseph et al., many of which are implicated in amino acid, carbohydrate, nucleotide and energy metabolism as well as the biosynthesis of cofactors and vitamins (Joseph et al., 2011); the implication being that genes with a role in pathogenicity will exhibit high rates of recombination (Joseph et al., 2011, Didelot and Maiden, 2010, Hao et al., 2011). All of these genes were also part of the Lineage 5 cgMLST, 329 of which containing *p*-distance values equal to or below *p*-0.002 (Fig. 3) and including 31 loci which were identical between all of the isolates in this study (Supplementary Table 1). It has been suggested that loci involved in key metabolic functions may be subject to selection resulting in particular combinations of gene alleles affecting the ability for meningococci to transmit among carriers (transmission fitness), in turn leading to allele sets associated with clonal complexes (Buckee et al., 2008). Comparison of the 1,752 core loci resulted in cgMLST allelic profiles which clustered isolates into three distinct sub-lineages (Fig. 2B), consistent with these containing a genome-wide allelic structure. This indicates that, even over a period of four decades and across the globe, these core genes had remained conserved within this clonal complex.

A total of 41 genes exhibited diversity levels comparable to many vaccine antigens (Supplementary Table 2), and many of these were outer membrane proteins and therefore subject to immune selection. However, diversity would have been expected to be associated with time, progressing over the course of the epidemic. In contrast, isolates from all four decades clustered together indicating a universal global and temporal gene pool. The identification of a star phylogeny, with sub-lineages located on short branches emanating from an internal node, is consistent with a recent population expansion originating from a founding event prior to 1969 (when the outbreak was first described). Significant phylogenetic diversification, evidenced by the much longer branches within each sub-lineage, ensued with distinct localised outbreaks, e.g. Cuba, Brazil, Norway, the United States and Chile. Taken together, these data indicate that the ST-32 serogroup B *N. meningitidis* pandemic had not been the result of the emergence of a single invasive “clone” followed by spread but, rather, had been the result of multiple distinct localised outbreaks. Thus, a rise in the incidence of ST-32/ET-5 meningococcal disease had possibly been the result of local host populations becoming colonised by meningococci expressing a previously unseen antigenic repertoire followed by a decline in disease several years later once host immunity had increased.

In using a gene-by-gene approach combined with Prokka, genes new to the Lineage 5 genome were discovered. These included the iron receptor complex HpuAB, type IV secretory systems (T4SS) as well as a gonococcal conjugative plasmid. HpuAB receptors enable the acquisition of iron from haemoglobin–haptoglobin and provide meningococci with an additional means of acquiring this essential nutrient. A variable distribution of this receptor has been described with HpuAB more prevalent among isolates associated with carriage while hyper-invasive clonal complexes were found to contain both HpuAB and HmbR, the latter involved in acquisition of iron from haemoglobin (Tauseef et al., 2011). Nine of the ST-32 isolates contained the HpuAB receptor and this appeared to be more frequent among sub-lineage 5.3 isolates, including all 3 of the Cuban isolates. The high *p*-distance values observed for all of the iron acquisition receptors is consistent with these systems eliciting a strong immune response and being subject to selective pressure (Fig. 3 and Supplementary Table 3).

Isolate SH-78 contained a gonococcal genetic island (GGI) closely related to Mc GGI-4 previously detected in a serogroup W ST-22 isolate (Woodhams et al., 2012) with a further seven isolates containing a *virB*-like operon. Approximately 80% of gonococci contain a GGI with the frequency of horizontal genetic transfer increased 500-fold in gonococcal isolates secreting DNA via these systems (Ramsey et al., 2011). Both of these elements are T4SS which are ancestrally related to bacterial conjugation machines mediating the transfer of DNA and proteins (Christie et al., 2005). Small local meningococcal disease outbreaks have been described occurring in men who have sex with men among whom the incidence of gonococcal infections is high and who represent a core group of individuals that have contributed to the spread of antimicrobial-resistant *N. gonorrhoeae* (Marcus et al., 2013, Lewis, 2013). The identification of an isolate containing a gonococcal conjugative plasmid is consistent with genetic exchange occurring between meningococci and gonococci, in spite of their ecological differences. T4SS among meningococci, if functional, will further encourage horizontal genetic transfer thereby promoting adaption of the *Neisseria* population in changing host environments. In addition, GGI have not been detected in commensal *Neisseria* to-date, with the distribution of the *virB*-like T4SS as yet unknown. It is thus possible that T4SS variants may be present in commensal *Neisseria* species.

In conclusion, this study provides a gene-by-gene account of genomic data from a collection of isolates belonging to the ST-32 clonal complex which had been a prominent cause of serogroup B meningococcal disease. In using a gene-by-gene approach, the biological significance associated with allelic variation was determined enabling genes essential or accessory to a clonal complex to be identified. Serogroup B meningococcal disease remains endemic in many parts of the world with the availability of a comprehensive serogroup B vaccine still to be developed. The analysis of a collection of isolates belonging to a clonal complex which had been a prominent cause of serogroup B meningococcal disease during a period of high disease incidence provides an invaluable tool for exploring the evolution of a hyper-invasive lineage and how surface exposed antigens, such as those included in vaccine formulations, may diversify over time.

## Funding

This work was funded by the 10.13039/501100004074Wellcome Trust (087622/Z/08/2) and NIPH. These funding sources were not involved in the manuscript design, data analysis or interpretation. DC, MCJM, JEB and OBH had full access to all of the data in the study and had final responsibility for the decision to submit for publication.

## Authors' Contributions

DAC and MCJM designed the study. OBH interpreted the data, performed the literature search, made the figures and wrote the manuscript. JEB undertook the bioinformatic analysis. DAC, MJCM and JEB commented on the manuscript.

## Conflicts of Interest

The authors declared no conflicts of interest.
