# Supplementary material for: Genomic Analysis of the Evolution and Global Spread of Hyper-invasive Meningococcal Lineage 5
Source: eBioMedicine. 2015 Jan 13;2(3):234–43. doi: 10.1016/j.ebiom.2015.01.004 (PMC4430826; doi:10.1016/j.ebiom.2015.01.004)
Supplement: Supplementary file 1 — Supplementary material. [file mmc1.docx]

**Appendix: Web supplementary information**

**Supplementary Table 1. Identical loci between isolates (214 loci)**

| **NMB Locus** | **NEIS designation** | **Gene (bp)** | **Product** |
| --- | --- | --- | --- |
| **Amino Acid Biosynthesis (5)** | | | |
| NMB0307 | NEIS1874 | *aroG* (1056) | 2-keto-3-deoxy-D-arabino-heptulosonate-7-phosphate synthase I alpha (EC 2.5.1.54) |
| NMB0440 | NEIS1721 | 873bp | prephenate dehydrogenase |
| NMB0940^b^ | NEIS0918 | *metX* (1140) | homoserine O-acetyltransferase |
| NMB1576 | NEIS1496 | *ilvH* (492) | acetolactate synthase 3 regulatory subunit |
| NMB1813^a^ | NEIS0407 | *aroK* (513) | shikimate kinase |
| **Purines, pyrimidines, nucleosides and nucleotides (3)** | | | |
| NMB1252 | NEIS1152 | *purM* (1035) | phosphoribosylaminoimidazole synthetase |
| NMB1307 | NEIS1244 | *ndk* (426) | nucleoside diphosphate kinase |
| NMB1709 | NEIS1624 | *thyA* (795) | thymidylate synthase |
| **Fatty Acid and phospholipid metabolism (1)** | | | |
| NMB1588^a, b^ | NEIS1509 | *pgsA* (564) | CDP-diacylglycerol--glycerol-3-phosphate 3-phosphatidyltransferase |
| **Biosynthesis of cofactors, prosthetic groups and carriers (9)** | | | |
| NMB0261^a^ | NEIS0255 | *ispA* (879) | Octaprenyl-diphosphate synthase (EC 2.5.1.-) / Dimethylallyltransferase (EC 2.5.1.1) / Geranyltranstransferase (farnesyldiphosphate synthase) (EC 2.5.1.10) / Geranylgeranyl pyrophosphate synthetase (EC 2.5.1.29) |
| NMB0310 | NEIS1871 | 429bp | Protoporphyrinogen IX oxidase, novel form, HemJ (EC 1.3.-.-) |
| NMB0576^a, b^ | NEIS0518 | *hemA* (1248) | glutamyl-tRNA reductase |
| NMB0684 | NEIS0635 | *ribH* (477) | 6,7-dimethyl-8-ribityllumazine synthase |
| NMB0693^b^ | NEIS0644 | *folC* (1275) | bifunctional folylpolyglutamate synthase/dihydrofolate synthase |
| NMB0718 | NEIS0669 | *hemH* (1083) | ferrochelatase |
| NMB1146^a^ | NEIS1087 | *bioB-1* (1053) | biotin synthetase |
| NMB1254 | NEIS1155 | *ribA* (594) | GTP cyclohydrolase II |
| NMB1616^b^ | NEIS1538 | *thiD* (807) | phosphomethylpyrimidine kinase |
| **Energy and Central Intermediary Metabolism (22)** | | | |
| NMB0123^a^ | NEIS0115 | 252bp | 4Fe-4S ferredoxin, iron-sulfur binding |
| NMB0241^a^ | NEIS0237 | *nuoA* (357) | NADH ubiquinone oxidoreductase chain A (EC 1.6.5.3) |
| NMB0243^a, b^ | NEIS0239 | *nuoC* (594) | NADH-ubiquinone oxidoreductase chain C (EC 1.6.5.3) |
| NMB0245^a, b^ | NEIS0241 | *nuoE* (474) | NADH-ubiquinone oxidoreductase chain E (EC 1.6.5.3) |
| NMB0246^a, b^ | NEIS0242 | *nuoF* (1302) | NADH-ubiquinone oxidoreductase chain F (EC 1.6.5.3) |
| NMB0340 | NEIS1830 | *gloA* (417) | Lactoylglutathione lyase (EC 4.4.1.5) |
| NMB0575^a^ | NEIS0517 | *gcvH* (387) | glycine cleavage system protein H |
| NMB0951^b^ | NEIS0928 | *sdhB* (708) | succinate dehydrogenase iron-sulfur subunit |
| NMB0959 | NEIS0935 | *sucC* (1167) | succinyl-CoA synthetase subunit beta |
| NMB0960 | NEIS0936 | *sucD* (891) | succinyl-CoA synthetase subunit alpha |
| NMB1134^a^ | NEIS1073 | *fdx-1* (342) | ferredoxin, 2Fe-2S type |
| NMB1158^a^ | NEIS1097 | 657bp | nickel-dependent hydrogenase, b-type cytochrome subunit |
| NMB1159^a, b^ | NEIS1098 | 891bp | NAD(P)HX dehydratase |
| NMB1285^b^ | NEIS1220 | *eno* (1287) | phosphopyruvate hydratase |
| NMB1287 | NEIS1222 | 291bp | ferredoxin |
| NMB1304^b^ | NEIS1241 | 1137bp | alcohol dehydrogenase |
| NMB1324^a^ | NEIS1261 | *trxB* (951) | thioredoxin reductase |
| NMB1474^a^ | NEIS1411 | 210bp | 4-oxalocrotonate tautomerase |
| NMB1516 | NEIS1445 | *fixS* (189) | FixS protein |
| NMB1584 | NEIS1504 | 870bp | 3-hydroxyacid dehydrogenase |
| NMB1940 | NEIS1912 | *atpB* (867) | ATP synthase F0F1 subunit A |
| NMB2154 | NEIS2132 | *etfA* (936) | electron transfer flavoprotein subunit alpha |
| **Transport and Binding (4)** | | | |
| NMB0557 | NEIS0498 | *erpA* (339) | probable iron-binding protein from the HesB, IscA, SufA family |
| NMB1199^b^ | NEIS1099 | *typA* (1812) | GTP-binding protein TypA |
| NMB1612^a^ | NEIS1533 | 807 | amino acid ABC transporter substrate-binding protein |
| NMB1728^a^ | NEIS1648 | *exbD* (435) | biopolymer transport protein |
| **DNA metabolism (13)** | | | |
| NMB0262^a, b^ | NEIS0256 | *xseB* (225) | Exodeoxyribonuclease VII small subunit (EC 3.1.11.6) |
| NMB0263^a^ | NEIS0257 | *engC* (924) | Ribosome small subunit-stimulated GTPase |
| NMB0689 | NEIS0640 | *greB* (492) | transcription elongation factor |
| NMB0725^a^ | NEIS2377 | 1059bp | modification methylase HgaI-1 |
| NMB0727^a^ | NEIS2379 | 651bp | N-6 adenine-specific DNA methylase |
| NMB0769 | NEIS0722 | 978bp | DNA polymerase III subunit delta' |
| NMB1222^b^ | NEIS1116 | *ung* (660) | uracil-DNA glycosylase |
| NMB1237 | NEIS1137 | *recR* (621) | recombination protein |
| NMB1290^a^ | NEIS2442 | 1014bp | C-5 cytosine-specific DNA-methylase |
| NMB1322 | NEIS1259 | 303bp | primosomal replication protein |
| NMB1514^b^ | NEIS1443 | *dnaQ-2* (735) | DNA polymerase III subunit epsilon |
| NMB2048 | NEIS2028 | *ligA-2* (825) | DNA ligase |
| NMB2082 | NEIS2061 | *exoA* (780) | exodeoxyribonuclease |
| **Transcription (2)** | | | |
| NMB0126^a^ | NEIS0118 | *nusG* (537) | Transcription antitermination protein |
| NMB1499^a^ | NEIS1430 | *rph* (729) | ribonuclease PH |
| **Protein synthesis and Fate (23)** | | | |
| NMB0144 | BACT000052 | *rplW* (315) | 50S ribosomal protein L23 |
| NMB0145^b^ | BACT000031 | *rplB* (834) | 50S ribosomal protein L2 |
| NMB0146 | BACT000019 | *rpsS* (279) | 30S ribosomal protein S19 |
| NMB0147 | BACT000051 | *rplV* (330) | 50S ribosomal protein L22 |
| NMB0149 | BACT000045 | *rplP* (417) | 50S ribosomal protein L16 |
| NMB0150 | BACT000058 | *rpmC* (192) | 50S ribosomal protein L29 |
| NMB0153 | BACT000053 | *rplX* (324) | 50S ribosomal protein L24 |
| NMB0160^a^ | BACT000059 | *rpmD* (186) | 50S ribosomal protein L30 |
| NMB0164 | BACT000065 | *rpmJ1* (114) | 50S ribosomal protein L36 |
| NMB0169 | BACT000046 | *rplQ* (369) | 50S ribosomal protein L17 |
| NMB0324^a^ | BACT000056 | *rpmA* (273) | 50S ribosomal protein L27 |
| NMB0325^a^ | BACT000050 | *rplU* (309) | 50S ribosomal protein L21 |
| NMB0722 | BACT000064 | *rpmI* (198) | 50S ribosomal protein L35 |
| NMB0723 | BACT000049 | *rplT* (360) | 50S ribosomal protein L20 |
| NMB1321 | BACT000018 | *rpsR* (231) | 30S ribosomal protein S18 |
| NMB1950^a^ | BACT000021 | *rpsU* (213) | 30S ribosomal protein S21 |
| NMB0125^a^ | NEIS0117 | *secE* (279) | Preprotein translocase subunit (TC 3.A.5.1.1) |
| NMB0721^a^ | NEIS0673 | *infC* (468) | translation initiation factor IF-3 |
| NMB1298 | NEIS1235 | *rsuA* (693) | ribosomal small subunit pseudouridine synthase A |
| NMB1312 | NEIS1248 | *clpP* (531) | ATP-dependent Clp protease proteolytic subunit |
| NMB1332 | NEIS1270 | *prc* (1485) | carboxy-terminal peptidase |
| NMB1587^a^ | NEIS1508 | 1104bp | protease |
| NMB2083 | NEIS2062 | *cysS* (1422) | cysteinyl-tRNA synthetase |
| **Regulatory Functions (4)** | | | |
| NMB1302 | NEIS1239 | *ihfB* (315) | integration host factor subunit beta |
| NMB1303 | NEIS1240 | 408bp | MerR family transcriptional regulator |
| NMB1585^a^ | NEIS2454 | 432bp | MarR family transcriptional regulator |
| NMB1711^a, b^ | NEIS1629 | 780bp | GntR family transcriptional regulator |
| **Cell Envelope (4)** | | | |
| NMB0332^b^ | NEIS1839 | *pilD* (861) | Leader peptidase (Prepilin peptidase) (EC 3.4.23.43) / N-methyltransferase (EC 2.1.1.-) |
| NMB1309 | NEIS1246 | *pilW* (762) | fimbrial biogenesis and twitching motility protein |
| NMB1809 | NEIS0411 | *pilN* (600) | pilN protein |
| NMB2090 | NEIS2070 | *gmhA* (594) | phosphoheptose isomerase |
| **Cellular Processes (14)** | | | |
| NMB0172 | NEIS0162 | *minE* (264) | Cell division topological specificity factor |
| NMB0248^a^ | NEIS2368 | 240bp | contains GIY-YIG catalytic domain which is involved in many cellular processes including DNA repair and recombination, transfer of mobile genetic elements and restriction of incoming foreign DNA |
| NMB0255^a^ | NEIS0249 | 576bp | Protein involved in cell division |
| NMB0342^b^ | NEIS1828 | *ispZ* (531) | Intracellular septation protein |
| NMB0691 | NEIS0642 | 498bp | colicin V production protein |
| NMB1023^b^ | NEIS1192 | 960bp | tRNA(cytosine32)-2-thiocytidine synthetase |
| NMB1076^a^ | NEIS1040 | *had* (669) | DnaA regulatory inactivator |
| NMB1105^a^ | NEIS2422 | 378bp | transaldolase (EC 2.2.1.2) |
| NMB1210 | NEIS2434 | 468bp | toxin-activating protein |
| NMB1238 | NEIS1138 | 1539bp | peptidyl-prolyl cis-trans isomerase-like protein |
| NMB1328^b^ | NEIS1267 | *trmB* (717) | tRNA (guanine-N(7)-)-methyltransferase (EC 2.1.1.33) |
| NMB1504 | NEIS1433 | *scpA* (855) | segregation and condensation protein A |
| NMB2091 | NEIS2071 | 609bp | hemolysin |
| NMB2095 | NEIS2075 | 375bp | adhesin complex protein |
| **Unknown/Hypothetical Proteins (110)** | | | |
| NMB0037 | NEIS0014 | *phnA* (330) | Alkylphosphonate utilization operon protein |
| NMB0060^a^ | NEIS0044 | 2019bp | oligopeptide transporter |
| NMB0087 | NEIS0072 | 198bp | hypothetical protein |
| NMB0135^a^ | NEIS2366 | 150bp | hypothetical protein |
| NMB0215^a^ | NEIS0207 | 567bp | Integral membrane protein |
| NMB0222^a^ | NEIS0222 | 474bp | hypothetical protein |
| NMB0237^a^ | NEIS0233 | 360bp | putative immunity protein. Proteins containing this domain are present in bacterial polymorphic toxin systems as an immediate gene neighbour of the toxin gene. |
| NMB0239^a^ | NEIS0235 | 621bp | conserved domain protein |
| NMB0247^a^ | NEIS0243 | 453bp | hypothetical protein |
| NMB0252^a^ | NEIS2369 | 708bp | hypothetical protein |
| NMB0266^a^ | NEIS0260 | 327bp | hypothetical protein |
| NMB0306^a^ | NEIS1875 | 195bp | hypothetical protein |
| NMB0311 | NEIS1870 | 246bp | hypothetical protein |
| NMB0404 | NEIS1764 | 321bp | hypothetical protein |
| NMB0477 | NEIS1671 | 537bp | hypothetical protein |
| NMB0482^a^ | NEIS0954=NEIS1665 | 279bp | hypothetical protein |
| NMB0484^a, b^ | NEIS0952=NEIS1663 | 198bp | hypothetical protein |
| NMB0500^a^ | NEIS2371 | 378bp | hypothetical protein |
| NMB0510^a^ | NEIS0449 | 264bp | hypothetical protein |
| NMB0538 | NEIS0477 | 510bp | hypothetical protein |
| NMB0562 | NEIS0503 | 216bp | hypothetical protein |
| NMB0571 | NEIS0513 | 360bp | hypothetical protein |
| NMB0654^a^ | NEIS0598 | 318bp | hypothetical protein |
| NMB0659^a^ | NEIS0607 | 402bp | hypothetical protein |
| NMB0673 | NEIS0622 | 588bp | hypothetical protein |
| NMB0674 | NEIS0623 | 183bp | hypothetical protein |
| NMB0685 | NEIS0636 | 324bp | hypothetical protein |
| NMB0780^a^ | NEIS2382 | 441bp | hypothetical protein |
| NMB0783^b^ | NEIS0735 | 480bp | hypothetical protein |
| NMB0817^a^ | NEIS2384 | 384bp | hypothetical protein |
| NMB0818^a^ | NEIS2385 | 411bp | hypothetical protein |
| NMB0897^a^ | NEIS0837 | 381bp | hypothetical protein |
| NMB0902^a^ | NEIS0843 | 192bp | hypothetical protein |
| NMB0903^a^ | NEIS0844 | 294bp | hypothetical protein |
| NMB0904^a^ | NEIS2394 | 261bp | hypothetical protein |
| NMB0905^a^ | NEIS0845 | 216bp | hypothetical protein |
| NMB0906^a^ | NEIS0846 | 855bp | hypothetical protein |
| NMB0908^a^ | NEIS0848 | 201bp | hypothetical protein |
| NMB0909^a^ | NEIS0849 | 525bp | hypothetical protein |
| NMB0910^a^ | NEIS0850 | 717bp | hypothetical protein |
| NMB0912^a^ | NEIS2395 | 627bp | hypothetical protein |
| NMB0913^a^ | NEIS0879 | *pemK* (348) | plasmid toxin PemK protein |
| NMB0914^a^ | NEIS0880 | *pemI* (237) | plasmid-related PemI protein |
| NMB0915^a^ | NEIS0881 | 492bp | hypothetical protein |
| NMB0917^a^ | NEIS0882 | 369bp | hypothetical protein |
| NMB0918^a^ | NEIS2396 | 132bp | hypothetical protein |
| NMB0958^b^ | NEIS0934 | 288bp | hypothetical protein |
| NMB0972^a^ | NEIS0952=NEIS1663 | 198bp | hypothetical protein |
| NMB0974^a^ | NEIS0954=NEIS1665 | 279bp | hypothetical protein |
| NMB0989^a^ | NEIS0969 | 165bp | hypothetical protein |
| NMB1002^a^ | NEIS0993 | 876bp | hypothetical protein |
| NMB1004^a^ | NEIS0996 | 246bp | hypothetical protein |
| NMB1007^a^ | NEIS2399 | 372bp | hypothetical protein |
| NMB1008^a^ | NEIS2400 | 624bp | hypothetical protein |
| NMB1009^a^ | NEIS2401 | 195bp | hypothetical protein |
| NMB1016 | NEIS1007 | 606bp | hypothetical protein |
| NMB1025^a^ | NEIS1190 | 363bp | hypothetical protein |
| NMB1035 | NEIS1178 | 255bp | hypothetical protein |
| NMB1048^a^ | NEIS1163 | 1470bp | hypothetical protein |
| NMB1059^a^ | NEIS1021 | 219bp | hypothetical protein |
| NMB1073^b^ | NEIS1037 | 1131bp | hypothetical protein |
| NMB1086^a^ | NEIS0970 | 237bp | hypothetical protein |
| NMB1088^a^ | NEIS0973 | 171bp | hypothetical protein |
| NMB1090^a^ | NEIS2409 | 267bp | hypothetical protein |
| NMB1098^a^ | NEIS2416 | 1062bp | Mu-like prophage FluMu I protein |
| NMB1101^a^ | NEIS2418 | 423bp | hypothetical protein |
| NMB1102^a^ | NEIS2419 | 594bp | hypothetical protein |
| NMB1103^a^ | NEIS2420 | 198bp | hypothetical protein |
| NMB1112^a^ | NEIS2429 | 348bp | bacteriophage protein GP46 |
| NMB1117^a^ | NEIS2430 | 303bp | hypothetical protein |
| NMB1120^a^ | NEIS1060 | 297bp | hypothetical protein |
| NMB1133^a, b^ | NEIS1072 | 762bp | hypothetical protein |
| NMB1138^a^ | NEIS1077 | 402bp | hypothetical protein |
| NMB1221^a^ | NEIS1115 | 408bp | hypothetical protein |
| NMB1236 | NEIS1136 | 279bp | hypothetical protein |
| NMB1253^b^ | NEIS1154 | 678bp | hypothetical protein |
| NMB1286 | NEIS1221 | 279bp | hypothetical protein |
| NMB1293 | NEIS1229 | 327bp | hypothetical protein |
| NMB1306 | NEIS1243 | 1152bp | hypothetical protein |
| NMB1308^b^ | NEIS1245 | 1095bp | hypothetical protein |
| NMB1369 | NEIS1304 | 555bp | hypothetical protein |
| NMB1444 | NEIS1380 | 336bp | hypothetical protein |
| NMB1500^a^ | NEIS1431 | 465bp | hypothetical protein |
| NMB1502^a^ | NEIS2450 | 528bp | hypothetical protein |
| NMB1517 | NEIS1446 | 300bp | hypothetical protein |
| NMB1575 | NEIS1495 | 294bp | hypothetical protein |
| NMB1590^a, b^ | NEIS1512 | 336bp | hypothetical protein |
| NMB1598^a^ | NEIS2456 | 192bp | hypothetical protein |
| NMB1611 | NEIS1532 | 252bp | hypothetical protein |
| NMB1743^a, b^ | NEIS0952=NEIS1663 | 198bp | hypothetical protein |
| NMB1745^a^ | NEIS0954=NEIS1665 | 279bp | hypothetical protein |
| NMB1774^a^ | NEIS2472 | 378bp | hypothetical protein |
| NMB1781^a^ | NEIS0441 | 171bp | hypothetical protein |
| NMB1819^a^ | NEIS2473 | 156bp | hypothetical protein |
| NMB1884 | NEIS0337 | 324bp | hypothetical protein |
| NMB1941^a^ | NEIS1913 | 354bp | hypothetical protein |
| NMB1959 | NEIS1931 | 384bp | hypothetical protein |
| NMB1960 | NEIS1932 | 483bp | hypothetical protein |
| NMB2013^a^ | NEIS2481 | 645bp | hypothetical protein |
| NMB2015^a^ | NEIS1994 | 270bp | hypothetical protein |
| NMB2063 | NMB2044 | 225bp | hypothetical protein |
| NMB2080 | NEIS2059 | 531bp | hypothetical protein |
| NMB2081 | NEIS2060 | 345bp | hypothetical protein |
| NMB2085^a^ | NEIS2064 | 1050bp | hypothetical protein |
| NMB2089 | NEIS2069 | 348bp | hypothetical protein |
| NMB2110^a^ | NEIS2089 | 372bp | hypothetical protein |
| NMB2124^a^ | NEIS2100 | 129bp | hypothetical protein |
| NMB2142 | NEIS2121 | 867bp | hypothetical protein |
| NMB2143 | NEIS2122 | 747bp | hypothetical protein |
| NMB2150 | NEIS2128 | 570bp | hypothetical protein |

^a^: identified as a core recombinant gene by Hao *et al*; ^b^: identified as a core recombinant gene by Joseph *et al* [[1](#_ENREF_1),[2](#_ENREF_2)]

**Supplementary Table 2. Loci with *p-*distance values equal to or greater than 0.015 (41 loci)**

| **NMB Locus** | **NEIS designation*** | **Gene (bp)** | **Product** | ***p-*distance** | **number of polymorphic sites (nonsysnomymous)** |
| --- | --- | --- | --- | --- | --- |
| **Capsule biosynthesis and transport** | | | | | |
| NMB0074 | NEIS0058 | *ctrD* (651) | capsule polysaccharide export ATP-binding protein | 0.034 | 10 |
| **Purine Ribonucleotide biosynthesis** | | | | | |
| NMB0284 | NEIS0290 | *purB* (1371) | Adenylosuccinate lyase (EC 4.3.2.2) | 0.026 | 10 |
| **DNA Replication, recombination, repair and metabolism** | | | | | |
| NMB0453^b^ | NEIS1699 | *mutT* (810) | 8-oxo-7,8-dihydroguanosine triphosphate pyrophosphatase activity | 0.024 | 155 (65) |
| NMB0551 | NEIS0491 | *priA (2190)* | primosome assembly protein | 0.034 | 257 (59) |
| NMB1673^b^ | NEIS1591 | *tag (552)* | DNA-3-methyladenine glycosylase | 0.021 | 24 (7) |
| NMB1868^b^ | NEIS0351 | *xerC (918)* | tyrosine recombinase | 0.033 | 182 (54) |
| **Iron Acquisition** | | | | | |
| NMB0460 | NEIS1691 | *tbpB (2139)* | transferrin-binding protein B | 0.170 | 1353 (485) |
| NMB0461 | NEIS1690 | *tbpA (2748)* | transferrin-binding protein A | 0.025 | 328 (108) |
| NMB1540 | NEIS1468 | *lbpA (2838)* | lactoferrin-binding protein A | 0.015 | 309 (99) |
| NMB1541 | NEIS1469 | *lbpB (2337)* | lactoferrin-binding protein B | 0.061 | 859 (371) |
| NMB1668 | NEIS1586 | *hmbR (2379)* | haemoglobin receptor | 0.044 | 256 (75) |
| NMB1669^b^ | NEIS1587 | *hemO (636)* | Heme oxygenase associated with heme uptake | 0.024 | 65 (14) |
| NMB1988 | NEIS1963 | *fetA (frpB) (2184)* | enterobactin receptor | 0.038 | 332 (112) |
| NMB1989^b^ | NEIS1964 | *piuA (966)* | Iron compound ABC uptake transporter substrate-binding protein | 0.043 | 127 (23) |
| NMB1993^b^ | NEIS1968 | *fecE (760)* | Iron(III) dicitrate transport ATP-binding protein (TC 3.A.1.14.1) | 0.021 | 153 (52) |
| **Toxin/anti-toxin components** | | | | | |
| NMB0515 | NEIS0456 | 384bp | putatively associated with the function of NMB0516; located in an operon | 0.077 | 64 (25) |
| NMB0516 | NEIS0457 | 936bp | A predicted immunity protein with an all alpha-helical fold and a conserved proline residue. Proteins containing this domain are present in bacterial polymorphic toxin systems as an immediate gene neighbour of the toxin gene, usually containing a domain of the Tox-REAse-1 or Tox-REase-6 families. Implicated in endonuclease activity. (ref Zhang D et al 2012 Biology Direct 7:18) | 0.060 | 133 (48) |
| **Type IV pilus biogenesis** | | | | | |
| NMB0547 | NEIS0487 | *pilE (390)* | Type IV pilus biogenesis protein | 0.072 | 83 (38) |
| NMB0886 | NEIS0827 | *pilH (679)* | type IV pilus biogenesis protein | 0.025 | 131 (52) |
| NMB0887 | NEIS0828 | *pilI (627)* | type IV pilus biogenesis protein | 0.026 | 114 (49) |
| NMB0888 | NEIS0832 | *pilJ (1030)* | type IV pilus biogenesis protein | 0.039 | 260 (112) |
| NMB0890^b^ | NEIS0831 | *pilX (513)* | minor pilin | 0.019 | 64 (32) |
| **Vaccine components** | | | | | |
| NMB1429^b^ | NEIS1364 | *porA* (1221) | outer membrane protein | 0.020 | 105 (37) |
| NMB1870 | fHbp | *fHbp* (880) | factor H binding protein | 0.086 | 277 (112) |
| NMB1994 | NEIS1969 | *nadA (1224)* | adherence and invasion outer membrane protein (binds collagen,fibronectin and laminins,provides protection against antimicrobial polypeptides) | 0.020 | 32 (21) |
| NMB2039 | NEIS2020 | *porB (996)* | outer membrane protein | 0.025 | 90 (41) |
| NMB2132 | NEIS2109 | *nhba (1509)* | putative periplasmic protein | 0.049 | 385 (180) |
| **House-keeping, metabolism and Transport** | | | | | |
| NMB0552 | NEIS0493 | *nnrS (1074)* | NnrS protein; putative heme-Cu protein expression which is dependent on the transcriptional regulator, NnrR (NMB0380). This locus contains a premature stop codon in all isolates excluding those found in Group 4. | 0.045 | 192 (135) |
| NMB1051 | NEIS1015 | *abcZ (1911)* | ABC transporter | 0.016 | 106 (12) |
| NMB1543 = NMB1634 | NEIS0277 | 1215bp | probable phage replication protein | 0.022 | 253 (83) |
| NMB1606 | NEIS1526 | 1518bp | sigma-54 dependent response regulator | 0.017 | 141 (64) |
| NMB1634 = NMB1543 | NEIS0277 | 1216bp | replication initiation factor, probable phage replication protein | 0.033 | 262 (84) |
| NMB1846^b^ | NEIS0372 | 1080bp | Scaffold protein for [4Fe-4S] cluster assembly ApbC, MRP-like | 0.031 | 126 (25) |
| NMB1871^b^ | NEIS0348 | 678bp | putative molecular chaperone | 0.035 | 136 (40) |
| NMB1905 | NEIS0318 | *rnpA (366)* | Ribonuclease P protein component (EC 3.1.26.5) | 0.034 | 165 (65) |
| NMB2052 | NEIS2033 | *petB (1350)* | Ubiquinol--cytochrome c reductase, cytochrome B subunit (EC 1.10.2.2) | 0.029 | 178 (23) |
| **Hypothetical Proteins** | | | | | |
| NMB0992 | NEIS0978 | *hsf (1780)* | outer membrane protein (GNA992) | 0.019 | 178 (83) |
| NMB1480 | NEIS2447 | *162bp* | hypothetical protein | 0.022 | 18 (8) |
| NMB1917 | NEIS0306 | 478bp | conserved integral membrane protein | 0.022 | 149 (60) |
| NMB2130 | NEIS2108 | *297bp* | hypothetical protein | 0.076 | 79 (37) |
| **Other** | | | | | |
| NMB0700 | NEIS0651 | *igA (5448)* | IgA-specific serine endopeptidase | 0.019 | 434 (165) |

^b^: identified as a core recombinant gene by Joseph *et al* [[2](#_ENREF_2)]

**Supplementary Table 3. Loci with a variable distribution among isolates compared to MC58 and H44/76 (47)**

| **NMB Locus** | **NEIS designation** | **Gene (bp)** | **Product** | **isolates the locus is absent in** |
| --- | --- | --- | --- | --- |
| **Cellular Processes (7)** | | | | |
| NMB0047 | NEIS2486 | 285bp | contains GIY-YIG catalytic domain (see NEIS2368) | CU-83 and JP-86. |
| NMB0419 | NEIS1746 | 597bp | tetratricopeptide repeat family protein; mediates protein-protein interactions and the assembly of multiprotein complexes | JP-79. |
| NMB0647 | NEIS0586 | 1239bp | MafB-related protein | JP-86 |
| NMB1268 | NEIS1199 | 1116bp | Glycerate kinase (EC 2.7.1.31) | NO-85. |
| NMB1692 | NEIS2494 | 894bp | 2-keto-3-deoxy-D-arabino-heptulosonate-7-phosphate synthase I beta (EC 2.5.1.54) | CU-92A, CN-74, AU-93 and TH-80. |
| NMB1766 | NEIS2505 | 1014bp | Large exoproteins involved in heme utilization or adhesion | NZ-95, MA-94A, MA-94B, NO-99; identical allele in all remaining isolates. |
| NMB1994 | NEIS1969 | *nadA* (1089) | adherence and invasion outermembrane protein (binds collagen,fibronectin and laminins,provides protection against antimicrobial polypeptides) | NO-76 (H44/76) |
| **Unknown/Hypothetical Proteins (37)** | | | | |
| NMB0091 | NEIS0080 | 237bp | hypothetical protein | CA-96 and AR-94; identical allele in all remaining isolates |
| NMB0092 | NEIS0081 | 228bp | hypothetical protein | CA-96 and AR-94; identical allele in all remaining isolates |
| NMB0094 | NEIS0083 | 189bp | hypothetical protein | CA-96 and AR-94 |
| NMB0095 | NEIS0084 | 153bp | hypothetical protein | CA-96 and AR-94 |
| NMB0096 | NEIS0085 | 171bp | hypothetical protein | CA-96 and AR-94 |
| NMB0099 | NEIS0089 | 144bp | hypothetical protein | CA-96 and AR-94; identical allele in all remaining isolates |
| NMB0200 | NEIS0193 | 390bp | hypothetical protein | all isolates except UK-83, JP-86, SH-78, CN-74, CA-79, CA-78. |
| NMB0272 | NEIS0267 | 297bp | hypothetical protein | UK-87 |
| NMB0432 | NEIS2488 | *yfcA* (789) | Putative membrane protein | DE-85, AR-94 and ZA-88. |
| NMB0491 | NEIS2489 | 444bp | hypothetical protein | NZ-95, MA-94A, MA-94B, NO-99; identical allele in all remaining isolates. |
| NMB0498 | NEIS0445 | 510bp | hypothetical protein | TH-80; identical allele in all remaining isolates. |
| NMB0503 | NEIS2490 | 432bp | hypothetical protein | NO-76, NO-75 and TH-80. |
| NMB0507 | NEIS0453 | 489bp | hypothetical protein | NO-76, AR-94, NO-75 |
| NMB0508 | NEIS0454 | 261bp | hypothetical protein | NO-76, AR-94, NO-75 |
| NMB0644 | NEIS0587 | 465bp | hypothetical protein | JP-86, SH-78, CN-74, CA-79, AU-963 and TH-80; identical allele in all remaining isolates. |
| NMB0820 | NEIS2491 | 393 bp | hypothetical protein | ZA-85 and ZA-88 |
| NMB0864 | NEIS0803 | 291bp | hypothetical protein | SP-85B and NO-08 |
| NMB0865 | NEIS0801 | 474bp | hypothetical protein | all isolates except UK-83 and NO-85. |
| NMB1343 | NEIS2492 | 438bp | hypothetical protein | DE-85 |
| NMB1402 | NEIS2493 | 435bp | hypothetical protein | NL-84, CU-92A, CU-83, CA-96, NZ-95, AU-93, UK-87, US-96, MA-94A, MA-94B, CI-98 and NO-99; identical allele in all remaining isolates. |
| NMB1755 | NEIS2496 | 344bp | hypothetical protein | NZ-95, MA-94A, MA-94B, NO-99; identical allele in all remaining isolates. |
| NMB1756 | NEIS2497 | 195bp | hypothetical protein | NZ-95, MA-94A, MA-94B, NO-99; identical allele in all remaining isolates. |
| NMB1758 | NEIS2470 | 212bp | hypothetical protein | NZ-95, MA-94A, MA-94B, NO-99; identical allele in all remaining isolates. |
| NMB1759 | NEIS2498 | 1380bp | hypothetical protein | NZ-95, MA-94A, MA-94B, NO-99; identical allele in all remaining isolates. |
| NMB1760 | NEIS2499 | 506bp | hypothetical protein | NZ-95, MA-94A, MA-94B, NO-99; identical allele in all remaining isolates. |
| NMB1761 | NEIS2500 | 1254bp | hypothetical protein | NZ-95, MA-94A, MA-94B, NO-99; identical allele in all remaining isolates. |
| NMB1762 | NEIS2501 | 1787bp | hypothetical protein | NZ-95, MA-94A, MA-94B, NO-99; identical allele in all remaining isolates. |
| NMB1763 | NEIS2502 | 458bp | hypothetical protein | NZ-95, MA-94A, MA-94B, NO-99; identical allele in all remaining isolates. |
| NMB1764 | NEIS2503 | 315bp | hypothetical protein | NZ-95, MA-94A, MA-94B, NO-99; identical allele in all remaining isolates. |
| NMB1765 | NEIS2504 | 300bp | hypothetical protein | NZ-95, MA-94A, MA-94B, NO-99; identical allele in all remaining isolates. |
| NMB1784 | NEIS0438 | 354bp | hypothetical protein | TH-80; identical allele in all remaining isolates. |
| NMB1850 | NEIS0368 | 276bp | hypothetical protein | SP-85A; identical allele in all remaining isolates. |
| NMB1854 | NEIS2506 | 642bp | hypothetical protein | NL-85. |
| NMB2116 | NEIS2093 | 345bp | hypothetical protein | AU-93, NO-75; identical allele in all remaining isolates. |
| NMB2118 | NEIS2094 | 318bp | hypothetical protein | AU-93; identical allele in all remaining isolates. |
| NMB2120 | NEIS2508 | 402bp | hypothetical protein | AU-93; identical allele in all remaining isolates. |
| NMB1753 | NEIS2495 | 438bp | Virulence-associated protein D | NZ-95, MA-94A, MA-94B, NO-99; identical allele in all remaining isolates. |
| **Transport and Binding proteins (2)** | | | | |
| NMB0393 | NEIS2487 | *emrE* (336) | Ethidium bromide-methyl viologen resistance protein | JP-86; identical allele in all remaining isolates |
| NMB2008 | NEIS2507 | 1644bp | ABC transporter, ATP-binding protein-related protein | NO-85 and JP-79. |
| **Transcription (1)** | | | | |
| NMB0646 | NEIS0589 | 288bp | ribonuclease inhibitor barstar | JP-86 |

1. Hao W, Ma JH, Warren K, Tsang RS, Low DE, et al. (2011) Extensive genomic variation within clonal complexes of *Neisseria meningitidis*. Genome Biology and Evolution 3: 1406-1418.

2. Joseph B, Schwarz RF, Linke B, Blom J, Becker A, et al. (2011) Virulence evolution of the human pathogen *Neisseria meningitidis* by recombination in the core and accessory genome. PLoS One 6: e18441.
